# The cell‐specific roles of Nrf2 in acute and chronic phases of ischemic stroke

**DOI:** 10.1111/cns.14462

**Published:** 2023-09-16

**Authors:** George Fadoul, Milos Ikonomovic, Feng Zhang, Tuo Yang

**Affiliations:** ^1^ Department of Neurology University of Pittsburgh Pittsburgh Pennsylvania USA; ^2^ Pittsburgh Institute of Brain Disorders and Recovery University of Pittsburgh Pittsburgh Pennsylvania USA; ^3^ Department of Psychiatry University of Pittsburgh Pittsburgh Pennsylvania USA; ^4^ Geriatric Research Education and Clinical Center, VA Pittsburgh Healthcare System Pittsburgh Pennsylvania USA; ^5^ Department of Internal Medicine University of Pittsburgh Medical Center Pittsburgh Pennsylvania USA

**Keywords:** astrocytes, endothelial cells, ischemic preconditioning, MCAO, microglia, neurons, neuroprotection, oligodendrocytes, oxidative stress

## Abstract

Ischemic stroke refers to the sudden loss of blood flow in a specific area of the brain. It is the fifth leading cause of mortality and the leading cause of permanent disability. The transcription factor nuclear factor erythroid 2‐related factor 2 (Nrf2) controls the production of several antioxidants and protective proteins and it has been investigated as a possible pharmaceutical target for reducing harmful oxidative events in brain ischemia. Each cell type exhibits different roles and behaviors in different phases post‐stroke, which is comprehensive yet important to understand to optimize management strategies and goals for care for stroke patients. In this review, we comprehensively summarize the protective effects of Nrf2 in experimental ischemic stroke, emphasizing the role of Nrf2 in different cell types including neurons, astrocytes, oligodendrocytes, microglia, and endothelial cells during acute and chronic phases of stroke and providing insights on the neuroprotective role of Nrf2 on each cell type throughout the long term of stroke care. We also highlight the importance of targeting Nrf2 in clinical settings while considering a variety of important factors such as age, drug dosage, delivery route, and time of administration.

## 1 INTRODUCTION

Ischemic stroke is the main cause of disability in the United States and a significant cause of death globally.^1^ Every year, there are about 800,000 ischemic strokes in the United States, 600,000 of which are recurrent events.^2^ Globally, one of six people has had an ischemic stroke in their lifetime, and 14 million people experience a stroke each year.^3^ However, aside from reperfusion therapy, very few approaches are available clinically to protect the brain against ischemic injury or boost brain recovery.^4^ Despite many attempts to design drugs that can lessen neural damage after ischemic stroke, practically none have translated to successful phase III trials. Recently, major flaws in preclinical studies have been identified,^5^ reviving the opportunity to re‐test several failed approaches, such as neuronal protection against oxidative stress.

One of the most promising targets for ischemic stroke therapy is the nuclear factor erythroid 2‐related factor 2 (Nrf2). Nrf2 is a master transcription factor that regulates and maintains redox homeostasis. Ischemic injury with or without reperfusion is associated with excessive oxidative stress leading to DNA damage and cell death. Therefore, regulating redox levels might be a promising direction in the management of ischemic stroke. In addition, Nrf2 is involved in complex interactions with nuclear factor‐κB (NF‐κB), a major transcription factor that triggers a panel of inflammatory pathways, antagonizing NF‐κB at multiple levels to regulate neuroinflammation and prevent additional injury after stroke.^6^, ^7^

**FIGURE 1:**
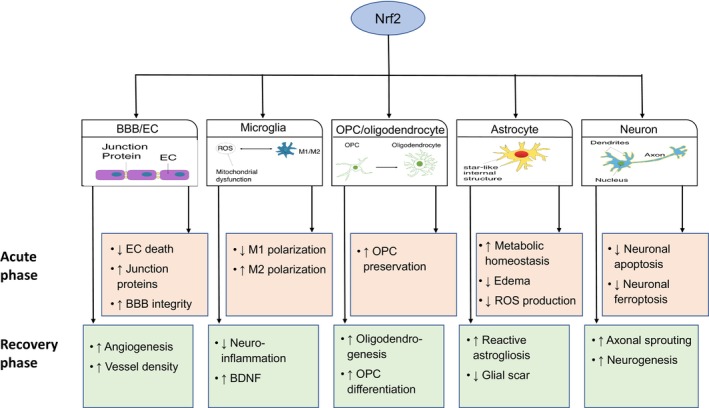
Cell‐specific effects of Nrf2 on ischemic stroke pathologies. Schematic image showing potential Nrf2 targets in acute and chronic recovery phases of ischemic stroke. BBB, blood–brain barrier; EC, endothelial cell; OPC, oligodendrocyte precursor cell; ROS, reactive oxygen species.

Cellular responses to ischemic stroke vary dramatically among different types of brain cells and between different phases of stroke. In this review, we provide an overview of the Nrf2 pathway in physiological and ischemic conditions and discuss the cell‐specific roles of Nrf2 in both acute and chronic phases of ischemic stroke (Figure 1).

## 2 OVERVIEW OF THE NRF2 PATHWAY AND ITS IMPLICATIONS IN ISCHEMIC STROKE

Human Nrf2 consists of 605 amino acids and 7 Nrf2‐ECH domains (Neh1‐7) and is a member of the cap'n’collar family of transcription factors.^3^ A nuclear localization signal is in charge of directing Nrf2 into the cell nucleus.^8^ Neh1 mediates the formation of Nrf2‐musculoaponeurotic fibrosarcoma (Maf) heterodimer and binds to the antioxidant response element (ARE) of the DNA.^9^, ^10^ Neh4 and Neh5 are mediating domains for Nrf2 interaction with coactivators such as cAMP response element‐binding protein (CREB)‐binding protein which enhances its ARE‐binding capacity.^11^ Neh7 domain binds to retinoic X receptor α, responsible for the competitive inhibition of Nrf2‐ARE binding.^12^ Neh2 and Neh6, which interact with Kelch‐like‐ECH‐associated protein 1 (Keap1) and β‐transducin repeat‐containing protein, respectively, mediate proteasomal degradation of Nrf2.^3^, ^13^

The expression level of Nrf2 is finely tuned by complex cellular mechanisms. Under normal conditions, Nrf2 is constantly produced and degraded via either the canonical, Keap1‐dependent pathway, or the alternative, Keap1‐independent pathway, forming a dynamic balance. Nrf2 is activated upon electrophile reaction and oxidative stress, which withholds Keap1 from sequestering Nrf2 and prevents its degradation.^14^ It has been shown that the Cys288 residue of Keap1 is critical for Keap1‐Nrf2 binding and dissociation.^15^ The alternative, Keap1‐independent pathway for Nrf2 degradation involves Nrf2 phosphorylation, by glycogen synthase kinase 3β (GSK3β). GSK3β can be directly adducted by electrophiles via its Cys299 residue,^15^ or indirectly regulated by the phosphatase and tensin homolog‐phosphoinositide 3‐kinase (PI3K)‐Akt axis.^13^ The inactivation of Nrf2 is regulated through its negative feedback loop. For example, the presence of ARE in the Keap1 gene promotor region results in Keap1 upregulation and Nrf2 degradation.^16^ Other factors such as the Keap1‐binding protein prothymosin and GSK3β‐mediated p‐Fyn also play a role in this negative feedback loop.^17^, ^18^ Additional details on Nrf2 structure, activation, inactivation, and degradation have been reviewed elsewhere.^19^

Over 200 Nrf2 downstream genes have been identified based on the presence of ARE. They are associated with a wide array of functions, such as redox balance, metabolism, inflammation, and proteostasis.^20^, ^21^ Nrf2 activation after ischemic stroke in animal models has been reported consistently by multiple groups. The timeline varies, possibly due to different experimental stroke models in different animal species. Details have been thoroughly reviewed elsewhere.^22^ In general, Nrf2 activation occurs hours after ischemic insult, and it lasts days to weeks before returning to baseline level.^23^, ^24^

The protective effects of Nrf2 depend on its temporal and spatial distribution after ischemic stroke. In this regard, Nrf2 activity has been evaluated using the ratio of nuclear to cytoplasmic Nrf2 levels after transient middle cerebral artery occlusion (MCAO) in rats.^25^ At 4 h after reperfusion, Nrf2 activity was significantly greater in the ipsi‐lesional hemispheres when compared to the contra‐lesional hemispheres. Interestingly, at 72 h after perfusion, Nrf2 activity at both hemisphere dropped below the baseline, with no difference observed between them. Levels of Nrf2 in the peri‐infarct region were significantly greater than in the core region at 24 and 72 h after reperfusion, most abundantly in glial fibrillary acidic protein (GFAP)‐positive reactive astrocytes,^25^ which is consistent with observation from our group.^26^, ^27^ Additionally, beneficial roles of Nrf2 in ischemic stroke have been demonstrated using Nrf2 activators or Nrf2 knockdown mice. Importantly, Nrf2 has critical neuroprotective roles in both acute and chronic phases after stroke, as reported previously by our group and others.^28^, ^29^, ^30^

## 3 CELL‐SPECIFIC ROLE OF NRF2 IN THE ACUTE PHASE

### 3.1 Neurons

Ischemic insults result in several forms of neuronal cell death. Necrosis owing to excitotoxicity is the predominant form in the ischemic core, while apoptosis mainly occurs in the penumbra.^31^ In the ischemic core, an abrupt cut‐off of ATP leads to a disruption in the Na^+^/K^+^ pump, and rapid K^+^ efflux results in transient hyperpolarization within seconds. Minutes later, the Na^+^/K^+^ pump fails and subsequently the disruption of the Ca^2+^/ATP pump and Na^+^/Ca^2+^ exchanger causes a massive efflux of K^+^ and an influx of Na^+^ and Ca^2+^, resulting in marked membrane depolarization. Excessive release of neurotransmitters such as glutamate ensues and further exacerbates neuronal membrane depolarization and energy consumption, resulting in an irreversible injury cascade, a process termed excitotoxicity.^32^ Excitotoxicity is a common way of neuronal death not only seen in acute brain ischemia but also present in other diseases such as chronic ischemia, trauma, and neurodegenerative disorders. Over the past several decades, multiple studies have reported the role of Nrf2 in the mitigation of neuronal excitotoxicity.^33^, ^34^, ^35^, ^36^ Nevertheless, excitotoxicity owing to acute neurovascular insult typically results in irreversible necrotic cell death within minutes, which is hardly preventable or rescuable. Although Nrf2 has been studied extensively, the mechanism(s) of its neuroprotective effects is not fully understood and requires further research. Recent research advances supported that Nrf2 serves as a key mechanism in many preconditioning approaches. For example, our studies demonstrated the essential role of Nrf2 in ischemic preconditioning (IPC) in neuronal cultures from both WT and Nrf2 knockout (KO) animals, where IPC‐afforded protection against oxygen–glucose deprivation (OGD) is abolished in Nrf2 KO neurons.^37^ This supports that Nrf2 plays a key role in protecting neurons against OGD in vitro. How exactly this may contribute to neuroprotection in vivo is not well understood.

#### 3.1.1 Nrf2 and apoptosis

A more desirable goal for neuroprotection in ischemic stroke is to mitigate neuronal apoptosis in the penumbra. Studies by our group and others consistently demonstrated the pivotal role of Nrf2 in reducing infarct volume and rescuing neuronal loss after ischemic stroke.^37^, ^38^, ^39^, ^40^ Mechanistically, sublethal ischemia in the penumbra leads to sublethal intracellular calcium accumulation, resulting in hyperpermeability of the mitochondria membrane. Apoptosis is mainly triggered by the leakage of mitochondrial content. For example, cytochrome C directly activates caspases, and reactive oxygen species (ROS) lead to DNA fragmentation and subsequent apoptotic cell death.^41^ Moreover, lipid peroxidation products such as 4‐hydroxynonenal could lead to DNA damage and subsequent apoptosis, and their scavenging with carbonyl reductase 1 or aldo‐keto reductase 1C15 could rescue neurons from DNA damage and ischemic injury in vivo and in vitro.^42^, ^43^, ^44^

Several studies defined the role of Nrf2 in mitochondrial function. For example, 3‐n‐butylphthalide protected against OGD‐induced PC12 (rat pheochromocytoma) neuronal cell death, and this was associated with Nrf2 activation, enhanced mitochondrial membrane potential, and increased activity of the mitochondrial respiratory chain complex I‐IV and ATPase.^45^ In a rat subarachnoid hemorrhage model, mitoquinone, a mitochondria‐target antioxidant, provided neuroprotection by reducing the release of Bax and cleaved Caspase‐3 from the mitochondria, which was completely abolished by ML385, an Nrf2 inhibitor.^39^ Mitochondrial protection and the necessity of Nrf2 activation in ischemic stroke need further validation. A more direct role of Nrf2 in neuroprotection is through mitigating oxidative stress by clearance of ROS, and alleviating excitotoxic injury which has been thoroughly reviewed elsewhere.^46^ Sublethal excitotoxicity in the penumbra predominantly leads to apoptosis rather than necrosis. It is worth noting that many studies only proved the association without further elucidating the causality. For example, in rats injected with monosodium glutamate, cyclooxygenase‐2 inhibitor flurbiprofen reduced hippocampal neuronal apoptosis, and this beneficial effect was associated with Nrf2 activation.^47^ A chalcone analog, 1‐(2,3,4‐trimethoxyphenyl)‐2‐(3,4,5‐trimethoxyphenyl)‐acrylketone, also inhibited glutamate‐induced cell apoptosis and along with Nrf2 activation it improved scopolamine‐induced cognitive impairment.^48^ In rats subjected to transient MCAO, carveol protected against oxidative stress (high lipid peroxidase) & cortical and striatal neuronal apoptosis via Nrf2 validated by injection of all‐trans retinoic acid (ATRA), an Nrf2 antagonist.^49^ This supported the critical role of Nrf2, although the off‐target effect of ATRA should be considered. In addition, tert‐butylhydroquinone (tBHQ) treatment reduced glutamate‐induced death of retinal ganglion cells in vitro, and retina thinning in vivo; these effects were associated with increased Nrf2 activation and reduced NF‐κB, and were abolished by Nrf2‐shRNA transfection^33^, ^50^ Studies by our and other groups also demonstrated that Nrf2 activators protect against OGD‐induced neuronal death in vitro.^26^, ^29^ Collectively, these studies support the direct role of Nrf2 in mitigating excitotoxicity in ischemic neurons. Mechanistically, this seems to be associated with the PI3K/Akt pathway, since rosmarinic acid and bone morphogenetic proteins alleviated stroke‐related neuronal apoptosis, which was associated with Akt activation, and was abolished when Akt phosphorylation was inhibited.^51^, ^52^ These observations support that Nrf2 plays a critical role in mitigating neuronal apoptosis, mainly through preserving mitochondrial function.

#### 3.1.2 Nrf2 and ferroptosis

Ferroptosis is a type of regulated cell death that is mechanistically and phenotypically unique from other forms of cell death. It is iron‐dependent, and lipid peroxide‐driven,^53^ which can be suppressed by iron chelators and lipophilic antioxidants. Ferroptosis is implicated in many neurological disorders such as traumatic brain injury (TBI) and in chronic neurodegenerative diseases including Alzheimer's disease and Parkinson's disease.^54^, ^55^ In the field of ischemic stroke, ferroptosis is more extensively studied in hemorrhagic stroke^56^, ^57^, ^58^, ^59^ as heme and iron are blood components. Indeed, ferroptosis was observed in neonatal rat hypoxic–ischemic brain injury models.^60^ A most recent study^61^ pooled several RNAseq databases from ischemic stroke patients and matched healthy controls and revealed significantly enriched ferroptosis‐related differentially expressed genes and the potential role of ferroptosis‐related biomarkers in ischemic stroke patients, indicating the critical role of ferroptosis in ischemic stroke.

Ferroptosis can be initiated by direct inhibition of glutathione peroxidase 4 (GPX4) and glutathione (GSH) depletion, and similarly, can be mitigated by GPX4 and GSH. Nrf2 controls a variety of essential enzymes involved in glutathione production and metabolism, such as glutathione synthetase (GSS), and a subunit of the cystine/glutamate transporter (xCT), which are crucial for GSH synthesis.^62^, ^63^ Additionally, GPX4 is a transcriptional target of Nrf2.^64^, ^65^ Therefore, Nrf2 could be a major regulator of lipid peroxidation and ferroptosis.^66^ Indeed, Nrf2 is critical for the anti‐ferroptotic effects of neuroprotective compounds such as vitexin,^67^ elabela,^68^ and inhaled propofol,^46^ and miRNA‐27a directly binds to the Nrf2 gene, inhibiting its expression which exacerbates ferroptosis.^68^ A limitation of ferroptosis research is that GSS and GPX4 are typically applied as markers for ferroptosis, but the potential involvement of other types of cell death has not been evaluated. Thus, such studies need to be interpreted with caution. Whether Nrf2 is essential for anti‐ferroptotis effects remains unsure, mainly due to a lack of specific ferroptosis markers.

### 3.2 Astrocytes

Astrocytes are the most abundant cell type in the central nervous system (CNS), presenting multiple supporting roles such as nutrient transportation, forming the blood–brain barrier (BBB), maintenance of ion balance, and participating in tissue repair.^69^ In primary cell cultures, astrocytes harbor more robust Nrf2 than neurons under physiological conditions.^70^ Nrf2 expression in astrocytes is detected in the acute phase of ischemic stroke. Studies from our group reported heme oxygenase‐1 (HO‐1) expression in astrocytes at 72 h post‐stroke in both the hippocampus and the cortex after transient MCAO in mice.^26^, ^27^ Another group used a rat ischemic stroke model and reported Nrf2 upregulation at 1d after stroke.^24^ Collectively, these studies suggest a positive effect of astrocytic Nrf2 in the acute phase after stroke. Indeed, astrocyte Nrf2 has been shown to be involved in several neuroprotective approaches, such as 11‐keto‐β‐boswellic acid or resveratrol.^71^, ^72^

Astrocyte Nrf2 exerts neuroprotection via a variety of mechanisms. Firstly, and most importantly, it helps to boost more effective metabolic activity. Astrocyte‐specific Nrf2 overexpression could help maintain neuronal metabolic homeostasis after mitochondrial complex II inhibition.^73^ In a resveratrol preconditioning model, Nrf2 was colocalized with the outer membrane of non‐synaptic, astrocytic mitochondria. Although no significant alterations were appreciated in the ratio of nuclear/mitochondrial subunits of oxidation phosphorylation (OXPHOS) or any of the complexes, Nrf2 KO mice had decreased formation of electron transport chain super complexes,^70^ which were defined as an oligomer of electron transport chain complexes harboring more efficient bioenergetics and producing less ROS.^74^ Secondly, astrocyte Nrf2 helps mitigate oxidative stress and subsequent cell death. For example, WD‐40 repeat protein 26 reduced H_2_O_2_‐mediated injury in primary human astrocyte cultures (U251‐MG, a glioblastoma cell line) associated with Nrf2/HO‐1 activation.^68^ In another study, icariside II protected primary astrocytes against OGD; this effect was associated with activation of the Nrf2 pathway and OXPHOS/NF‐κB/ferroptosis axis, which is dependent on Nrf2 as proved using Nrf2‐siRNA.^69^ Thirdly, astrocyte Nrf2 helps maintain the water and glutamate balance, as Nrf2 KO exhibited worsened edema and excitotoxicity.^70^ Last but not least, vascular endothelial growth factor (VEGF) production in astrocytes is dependent on Nrf2, as demonstrated in a study using Nrf2 KO mice.^71^

Astrocyte Nrf2 deserves to gain more attention in future studies. Mice overexpressing astrocyte‐specific Nrf2 have been used in studies of Alzheimer's disease,^75^ Parkinson's Disease,^76^ chronic hypoperfusion,^77^ and amyotrophic lateral sclerosis,^78^ and this could be a powerful tool for stroke research as well.

### 3.3 Oligodendrocytes

Studies related to Nrf2 and oligodendrocytes in the context of acute ischemic stroke are quite limited. In rat primary oligodendrocyte precursor cell (OPC) cultures subjected to OGD, miRNA‐146b‐5p overexpression prevented cell apoptosis in association with activation of Nrf2, supporting that Nrf2 might help maintain oligodendrocyte lineage homeostasis and prevent cell death.^79^ Nrf2 was also found to be critical for cell survival and metabolic activity in OPC cultures upon exposure to complex IV inhibitors.^80^ In experimental models of multiple sclerosis, Nrf2 was found to preserve oligodendrocytes and myelin.^81^, ^82^ These studies support that activation of Nrf2 in oligodendrocytes can enhance cell preservation and prevent cell apoptosis. Whether similar effects would be reproduced in ischemic stroke remains to be explored.

### 3.4 Microglia

Microglia are the main producers of proinflammatory cytokines which further exacerbate acute inflammation after ischemic stroke.^83^, ^84^, ^85^ Although acute inflammation is critical to clear up the dead tissue for subsequent brain repair, excessive neuroinflammation leads to secondary brain injury and tissue disruption, which hinders long‐term regeneration and reestablishment of function.^86^, ^87^ As a result, proper control of the extent and duration of neuroinflammation is required. As CNS resident myeloid cells, microglia are subclassified as resting, pro‐inflammatory (formerly known as M1), and anti‐inflammatory (formerly known as M2) phenotypes.^88^, ^89^ After stroke, M1 phenotype started to increase from day 3 onward and remained high until 7–14 days while the M2 phenotype peaked at day 3–5 and then subsided.^90^ It is important to note that resident microglia and infiltration macrophages are hard to differentiate after stroke, which is circumvented by most studies in the field and is beyond the scope of the present review. We, therefore, apply “microglia/macrophage” in the present review when these two cell types are undistinguishable in the referenced studies.

Studies have consistently reported that Nrf2 is associated with the inhibition of M1 polarization and enhancing M2 polarization in microglia/macrophages after stroke. For example, in a mouse permanent MCAO model, dexmedetomidine significantly improved stroke outcomes associated with enhanced M2 polarization in microglia/macrophages, which was abolished by Nrf2 inhibitor ML385, suggesting a critical role of Nrf2 in this process.^91^ In the transient MCAO model, Nrf2 proved to be essential in sevoflurane preconditioning as adeno‐associated virus (AAV)‐shNrf2‐abolished this protection.^92^ A similar association was also reported in the protection afforded by tanshinol borneol ester,^23^ non‐mitogenic fibroblast growth factor 1,^40^ and HP‐1c, a dual Nrf2/AMP‐activated protein kinase (AMPK) activator.^93^ In primary microglial cultures or BV2 cultures, the causal relationship between Nrf2 and M2 has been proven consistently, upon either OGD or lipopolysaccharide stimuli.^40^, ^91^, ^92^, ^93^ Thus, the effects of Nrf2 activation on microglia polarization may contribute to reducing the pro‐inflammatory cytokines and increasing anti‐inflammatory markers after brain insults including ischemic stroke.

It is worth mentioning that the M1/M2 classification is an oversimplification that fails to capture the full spectrum of microglia behavior in stroke^94^ and other neurodegenerative conditions. This classification has been derived primarily from in vitro cultures upon simple cytokine stimulus and it represents two rather extreme types of microglial activation. However, in vivo microglia display a complex spectrum with overlapping markers of both phenotypes, suggesting that microglia are highly flexible and highly dynamic cells with a complex role during stroke.^95^, ^96^, ^97^, ^98^

### 3.5 Endothelial cell (EC)/blood–brain barrier

Post‐stroke BBB disruption is a result of both direct ischemic injury to the ECs and indirect damage from oxidative stress and inflammation. Inflamed ECs allow leukocytes to travel across the BBB by interacting with the adhesion molecules such as E‐selectin and P‐selectin.^99^ Details on how BBB disruption happens and its consequences after stroke have been thoroughly reviewed elsewhere.^100^

Studies from our group and others consistently reported robust HO‐1 expression in the microvessels within the peri‐infarct region within 3 days after stroke, substantially more pronounced than in neurons or microglia.^15^, ^25^ Specifically, in rats subjected to hypoxia, Nrf2 binding to the ARE has been observed in the nuclear protein extracts from brain microvessel preparations as early as 1 h post hypoxic insult,^101^ suggesting a critical role of Nrf2 in microvessels or, more directly, the BBB. Indeed, many studies including ours consistently reported BBB protection by Nrf2. For example, EC and BBB protection was observed through direct Nrf2 modulators such as sulforaphane (Sfn), dimethyl fumarate (DMF), and others.^25^, ^102^, ^103^, ^104^ Similar findings have also been reported in experimental models of TBI.^105^

Mechanistically, Nrf2 protects against ischemic‐related EC death. For example, in primary mouse brain microvascular ECs, it was shown that Sfn reduced the cell death induced by OGD.^38^ PC and dichloroacetic acid‐afforded protection against OGD‐induced EC death and in vitro BBB disruption is dependent on Nrf2 as well.^38^, ^104^ Another important mechanism of BBB protection is that Nrf2 preserves the junction proteins, which are critical for BBB integrity. For example, DMF protects against OGD‐mediated zonula occludens‐1 (ZO‐1) dysfunction by preventing gap formation on the ZO‐1 membranous lining, and this effect is dependent on Nrf2 activation.^103^ In addition, studies from our group reported that Nrf2 could directly upregulate the expression of claudin 5 and cadherin 5 by enhancing their respective promoter activities.^15^, ^38^ It can be concluded that Nrf2 contributes to BBB protection after stroke, through lowering cell death and increasing cell junction proteins in ECs.

## 4 CELL‐SPECIFIC ROLE OF NRF2 IN THE CHRONIC RECOVERY PHASE

### 4.1 Neurons

The brain has the ability to change its network structure through growth, reorganization, and regeneration, termed neuroplasticity, as an intrinsic mechanism to facilitate recovery after stroke.^106^ Our most recent study using a mouse distal MCAO model reported that Sfn treatment alleviated long‐term axonal disintegration at 35‐day post‐stroke and this effect was dependent on Nrf2.^29^ A similar finding was also reported in a TBI model,^107^ further supporting the role of Nrf2 in neuronal protection and neuroplasticity.

Mechanistically, Nrf2 is critical for axonal sprouting and neurite outgrowth after stroke. In the distal MCAO model in vivo, we showed that Sfn administration after stroke resulted in Nrf2‐enhanced axonal sprouting which was abolished in the Nrf2 KO mice.^29^ Similar findings were noted in a spinal cord injury (SCI) model, where metformin promoted axonal growth after SCI in an Nrf2‐dependent manner, associated with reduced oxidative stress and improved mitochondrial function.^108^ Several in vitro studies also demonstrated the critical role of Nrf2 in neurite outgrowth. For example, in PC12 cell cultures, Akt overexpression activated Nrf2/ARE and promoted axonal growth,^109^ and in neuronal cultures subjected to hypoxic injury extracellular vesicles derived from human neural stem cells promoted elongation of neuronal axons along with Nrf2 activation.^110^ More direct evidence is needed to tell whether Nrf2 is also critical for neuronal axonal sprouting in stroke in vivo.

Another important mechanism of neural recovery after stroke is neurogenesis. In the adult brain, the two best‐described neurogenic niches are the adult neural stem and progenitor cells (NSPCs) which reside in the ventricular–subventricular zone (SZV) and the dentate gyrus.^111^, ^112^ Interestingly, reduced neural stem cell number and self‐renewal capacity were observed with increased age.^113^, ^114^ Cortical neurogenesis has also been reported, though at variable degrees which was closely related to the severity of ischemic injury.^115^, ^116^ Nrf2 has been reported to boost neurogenesis both in vivo and in vitro. Inducing Nrf2 overexpression with AAV in middle‐aged mice (11 months) improved cognitive and motor function, along with increased NSPC proliferation, self‐renewal, neurogenesis, and migration.^117^ Consistently, SVZ NSPC cultures show reduced Nrf2 expression with age. Nrf2 knockdown with siRNA reduced NSPC proliferation, and NSPCs from Nrf2 KO mice had lower survival and proliferation.^118^ Collectively, these findings suggest a critical role of Nrf2 in neurogenesis and support the idea that targeting Nrf2 is a promising therapy strategy to promote neurogenesis during chronic recovery from stroke.

### 4.2 Astrocytes

Astrocyte function in the chronic phase after stroke is complex. On one hand, reactive astrogliosis in the penumbra is beneficial by preventing the expansion of the ischemic core.^119^, ^120^ Inhibition of reactive astrogliosis by fluorocitrate 5 days after stroke exacerbated neurobehavioral deficits, suppressed neurovascular remodeling, and worsened stroke outcomes.^121^ On the other hand, severe gliosis led to glial scar formation, a maladaptive tissue reorganization pattern that prevents axonal regeneration and contributes to neurotoxicity and inflammation.^122^ For example, gliosis surrounding the ischemic core is not completely impermeable, and leakage from the core results in sustained liquefactive necrosis and neurodegeneration after stroke.^123^ Glial scar secretes inhibitory factors such as chondroitin sulfate proteoglycans which result in an environment inhibitory to axonal regeneration.^124^, ^125^

Several studies supported that Nrf2 is associated with reactive astrogliosis after stroke. tBHQ was able to promote reactive GFAP^+^ astrocytes proliferation in the peri‐infarct area from 7–28 days after MCAO, which was absent in the Nrf2 KO group, supporting that this upregulation of astrocytes proliferation is dependent on Nrf2.^39^ Similar findings were also reported in DMF and Korean red ginseng mediated Nrf2 activation, where reactive gliosis correlated well with levels of glutamine synthetase and aquaporin 4, a possible link to excitotoxicity and brain edema.^56^, ^126^ It has also been suggested that reactive astrogliosis relates to decreased GSK3β.^37^ Few studies have examined the effect of Nrf2 on glial scar formation. Nrf2 attenuates neuroinflammation which is a key mechanism contributing to a detrimental role of the glial scar. In addition, our recent study suggested that neurite sprouting after stroke is dependent on Nrf2, which may or may not be related to glial scar mitigation.^29^

In summary, evidence exists that Nrf2 promotes reactive astrogliosis. Given the complicated role of astrocytes in the chronic phase after stroke, whether Nrf2 mitigates glial scar formation is inconclusive.

### 4.3 Oligodendrocytes

Remyelination is part of white matter plasticity and repair after stroke, due to major white matter loss. Oligodendrogenesis and OPC differentiation into mature oligodendrocytes are important elements of remyelination. By far, few studies examined this aspect in the chronic phase after stroke. Our group recently reported the indispensable role of Nrf2 in Sfn‐mediated myelin preservation and oligodendrogenesis 35 days after ischemic stroke.^29^ The critical role of Nrf2 in oligodendrogenesis has also been reported after IPC^127^ and cuprizone‐mediated demyelination,^128^ and Nrf2 activator DMF‐induced differentiation in OPC cultures.^129^ It is very likely that Nrf2 enhances oligodendrogenesis and OPC differentiation, yet more work is needed to further elucidate OPC‐ or oligodendrocyte‐specific Nrf2 after ischemic stroke.

### 4.4 Microglia

Microglia activation can be sustained into the chronic phase, which is related to progressive neurodegeneration. For example, a unique CD11c^+^ microglia subtype was observed in relation to degenerative changes in the thalamus 28 days after ischemic stroke in mice. Knocking down of resident microglia, using an intracerebroventricular injection of lentiviral particles carrying shRNA targeting colony‐stimulating factor 1 receptor, prolonged survival, relieved neuroinflammation, and improved white matter remyelination at 3 weeks after MCAO in diabetic mice.^130^ Given the robust anti‐inflammatory property of Nrf2, it is expected that Nrf2 activators would attenuate neuroinflammation in the chronic phase of ischemic stroke which would lead to better outcomes. Indeed, this was reported using a dual AMPK/Nrf2 activator, and tanshinol borneol ester as well.^23^, ^93^ Cell type specificity of these responses needs to be elucidated in future studies.

In addition to their role in chronic neuroinflammation, microglia are important neurotrophic cells that could facilitate tissue repair, neuroplasticity, and recovery. During the recovery phase, microglia could produce local trophic gradients such as brain‐derived neurotrophic factor (BDNF), which promotes axonal regeneration^131^ and enhances synaptic efficacy.^132^ Indeed, knocking out of the BDNF specifically in microglia led to learning deficits and reduced synapse formation.^133^ Importantly, Nrf2 is a direct transcriptional activator of BDNF in cultured BV2 cells, through binding with the BDNF exon I promoter.^134^ After ischemic stroke in mice, Nrf2 activator bergenin enhanced BDNF expression in brain tissue.^135^

In summary, Nrf2 inhibits prolonged microglial activation and boosts BDNF expression in microglia in the chronic phase after stroke.

### 4.5 Endothelial cells

Angiogenesis is an important aspect of long‐term recovery post‐ischemia as it leads to the formation of new blood vessels. It occurs 3–4 days after stroke, predominantly at the penumbra, and its extent seems to correlate with the length of survival after stroke.^136^, ^137^, ^138^

tBHQ increased vessel density at d7, d14, and d28 post‐stroke in the peri‐infarct zone, and this effect was associated with higher VEGF levels in brain tissue at d7 and d14, and increased numbers of BrdU/CD31 double‐positive cells, a marker of newly generated ECs. Importantly, this increase was observed to a much lesser extent in the Nrf2 KO group, indicating a critical role of Nrf2 in angiogenesis.^39^ Similarly, in a rat MCAO model, corilagin increased CD34/dextran double‐positive ECs 7d after MCAO, which was partially abolished by Nrf2‐siRNA intrathecal administration.^100^ Such a causal relationship was also proved by the gain and loss of function of Nrf2 conducted through AAV and lentiviral interference in photothrombotic stroke.^139^ Nrf2 pathway activation is also observed to accompany epigallocatechin‐3‐gallate‐mediated angiogenesis 35 days post‐MCAO in mice.^140^ In cultured human brain microvascular ECs, quercetin promotes cell viability, migration and angiogenesis upon hypoxic injury, associated with Nrf2 activation.^141^ And in cultured mouse cerebral microvascular ECs (bEnd.3), knockdown of Nrf2 inhibited proliferation, migration, and tube formation after hypoxia, associated with downregulation of PI3K/Akt pathway.^142^ These findings consistently suggest a critical role of Nrf2 in angiogenesis after stroke.

An important point to consider is that angiogenesis after ischemic stroke may not always be beneficial. Indeed, pathological angiogenesis may further impede organ function. Interestingly, Nrf2 activation was observed in a mouse postnatal (P12‐17) oxygen‐induced retinopathy model, where Nrf2 KO mice showed impeded vascular regeneration and increased pathological neovascularization. Application of conditional KO mice where Nrf2 was knocked out in neurons, astrocytes, or ECs, respectively, revealed a critical role of neuronal and endothelial Nrf2 in regeneration.^143^ Unfortunately, stroke research mainly focuses on newly generated ECs without further examining their functioning. Future studies should focus more on vascular functioning such as the BBB and local perfusion, aside from simply describing EC regeneration and angiogenesis.

## 5 CLINICAL CONSIDERATIONS AND FUTURE PERSPECTIVES

Preclinical investigations supported the neuroprotective effects of Nrf2 in ischemic stroke. For clinical use, however, it will be important to optimize drug delivery, drug dose, and timing of administration. In addition, previously tested Nrf2 modulators need to be evaluated to determine optimal treatment protocol and to avoid side effects.^144^

It should be also emphasized that many other factors such as age could influence Nrf2 activation and its protective effects in vivo.^145^ Aging has been associated with Nrf2 dysfunction across multiple species and in different organs.^6^ The mechanisms responsible for this dysfunction involve miRNA, post‐translational protein modification, and impaired Nrf2‐Maf binding.^146^, ^147^ Indeed, (−)‐epicatechin reduced the activation and recruitment of microglia/macrophages at 7 days post‐stroke in young (2 months old) WT mice, but not in aged (12 months old) WT mice or aged Nrf2 KO mice,^148^ suggesting that sensitivity to Nrf2 activators decreases with age.

The potential benefits of using Nrf2 activators in the context of ischemic stroke are threefold. First, in high‐risk patients, pretreatment with Nrf2 activators could mimic IPC and establish tolerance to protect against a subsequent, major stroke. Second, activation of the Nrf2 pathway in the acute‐phase post‐stroke may reduce neuronal loss due to less inflammation and better protection of BBB and white matter. Third, activation of the Nrf2 pathway in the chronic phase post‐stroke could contribute to tissue repair and regeneration. Several Nrf2 pharmaceutical activators have been reviewed elsewhere,^149^, ^150^ and the most promising ones such as Sfn, sulforadex, curcumin, and resveratrol have undergone, or are undergoing, clinical trial testing for the treatment of various disorders.^151^ Notably, the majority of the Nrf2 activators have some off‐target effects. One possible solution to limit off‐target effects is to inject the drug in its inactive form, and then activate it once it crosses the BBB or when it is exposed to oxidative stress. This would mean that the bioactive component is released at the right time and in the right location.^152^

In conclusion, targeting Nrf2 is a promising strategy for the management of ischemic stroke. More in‐depth research is needed to further evaluate the benefits of cell‐specific and phase‐specific Nrf2 activation in providing optimal neuroprotection and better functional outcome after stroke.

## CONFLICT OF INTEREST STATEMENT

None.

## Data Availability

Data sharing not applicable to this article as no datasets were generated or analysed during the current study.
